# Emerging insights into the role of ferroptosis in the pathogenesis of autoimmune diseases

**DOI:** 10.3389/fimmu.2023.1120519

**Published:** 2023-03-30

**Authors:** Junyu Fan, Ting Jiang, Dongyi He

**Affiliations:** ^1^ Department of Rheumatology, Guanghua Hospital Affiliated to Shanghai University of Traditional Chinese Medicine, Shanghai University of Traditional Chinese Medicine, Shanghai, China; ^2^ Department of Rheumatology, Shanghai Guanghua Hospital of Integrated Traditional Chinese and Western Medicine, Shanghai, China; ^3^ Institute of Arthritis Research in Integrative Medicine, Shanghai Academy of Traditional Chinese Medicine, Shanghai, China

**Keywords:** ferroptosis, lipid metabolism, reactive oxygen species biology, iron homeostasis, immunity, autoimmune diseases

## Abstract

Ferroptosis, a novel type of regulated cell death mediated by iron-dependent lipid oxidation, was discovered a decade ago. Significant progress has been made in our knowledge of ferroptosis and immune dysfunction. This review covers recent advancements in the interaction of ferroptosis and the immune system, with an emphasis on autoimmune diseases. The critical regulators of ferroptosis are summarized in the context of reactive oxygen species biology, lipid metabolism, and iron homeostasis. The molecular crosstalk between ferroptosis and different immune cells is also highlighted. Future research is expected to yield new insights into the mechanisms governing ferroptosis and its potential therapeutic benefits in autoimmune diseases.

## 1 Introduction

Cell death and immunity are two essential biological activities that have been conserved throughout evolution to maintain homeostasis by means of intricate cellular and molecular interactions (1). The timely removal of cellular debris mediated by efferocytosis is critical to preventing the onset of autoimmune and inflammatory diseases. Meanwhile, exposing intracellular molecules to cells in the process of dying or that are already dead can induce adaptive immune responses, which are necessary to fight intracellular pathogens and tumor-associated antigens (1). Ferroptosis, an atypical type of cell death first reported by Dixon and colleagues in 2012 (2), is fundamentally different from other types of cell death, such as pyroptosis, apoptosis, autophagy, and necroptosis (3, 4). It is widely accepted that lipid peroxidation and iron metabolism are two essential elements of ferroptosis (5). Numerous studies show that an overabundance of iron promotes ferroptosis through the production of reactive oxygen species (ROS). The inhibition of glutathione peroxidase 4 (GPX4) or the depletion of glutathione (GSH) leads to ferroptosis by excessive lipid peroxidation and elevated intracellular lipid ROS. Furthermore, ROS damages membranes and causes cell death by attacking polyunsaturated fatty acids (PUFAs) in lipid membranes, generating high amounts of lipid peroxides. Small-molecule compounds, including RAS-selective lethal 3 (RSL3) and erastin, promote ferroptosis, whereas proteins such as liproxstatin-1 (Lip-1), ferrostatin-1 (Fer-1), and the iron chelator deferoxamine (DFO) exert opposite effects (6–9).

In recent years, considerable progress has been made in understanding ferroptosis on a molecular level since it appears to be the primary cause of several diseases and affords a range of pharmacologically tractable therapeutic intervention nodes (10). Alterations in the ferroptosis-regulating network also contribute to the development of autoimmune diseases. Therefore, this review aims to present emerging evidence on the molecular basis of ferroptosis and related functions in the immune system. We further attempt to discuss the pathogenic and therapeutic implications of ferroptosis in different autoimmune diseases to identify possible diagnostic targets and therapeutic options.

## 2 Core mechanisms of ferroptosis

Many studies have shed light on the inherent mechanisms that inhibit ferroptosis, the critical factors of iron metabolism that govern ferroptosis sensitivity, and the specified lipids that induce oxidation during ferroptosis. This has revolutionized our understanding of how ferroptosis affects diverse biological and pathological processes. Thus, in this section, we will focus on the importance of ferroptosis in the crosstalk of lipid metabolism, oxidative stress biology, and iron homeostasis.

### 2.1 Lipid metabolism

The peroxidation of certain lipids in the membrane is essential for ferroptosis. Free PUFAs were formerly thought to be the basis of ferroptosis due to their vulnerability to peroxidation caused by the weak C–H bonds between successive C=C double bonds. However, a recent study has shown that in order to lethally impact the peroxidation process, PUFAs must be activated and integrated into phospholipids (PLs), a form of membrane lipid, and that free fatty acids cannot trigger ferroptosis. Over the last decade, a major achievement in ferroptosis has been the discovery of specialized lipids that induce cell death and the identification of enzymes that promote integration into cell membranes.

The acyl-coenzyme A (CoA) synthetase long-chain family (ACSL) located on the mitochondrial outer membrane and endoplasmic reticulum (ER) can catalyze the transformation of fatty acids to acyl-CoAs (11). As intermediates in the lipid metabolic chain, acyl-CoAs are involved in fatty acid metabolism and membrane modifications. Five ACSL isoforms (ACSL1, ACSL3, ACSL4, ACSL5, and ACSL6) have been found in humans and rodents (12, 13). ACSL4 preferentially activates arachidonic acid by thioesterifying it with CoA, resulting in arachidonyl-CoA (14–16). Phosphatidylcholine (PC) is a major PL with a wide range of structural diversity in mammalian cell membranes (17). Four lysophatidylcholine acyltransferases (LPCATs), LPCAT1–4, participate in PC remodeling (18). The primary isoform, LPCAT3, is particularly important in lipoprotein production as it selectively catalyzes the insertion of acylated arachidonic acid into membrane phospholipids (12, 19). Thus, deletion of ACSL4 and LPCAT3 is thought to suppress ferroptosis by restricting the membrane-resident pool of oxidation-sensitive fatty acids.

Studies conducted on the haploid cell line KBM7 through insertional mutagenesis indicate that the inactivation of LPCAT3 and ACSL4 allows cells to become resilient against RSL3 and ML162, two GPX4 inhibitors (20). Additionally, a CRISPR suppression screen and ferroptosis-resistant cell line investigation revealed the deactivation of ACSL4 as a critical mechanism for inhibiting ferroptosis in various conditions. Conversely, ACSL4 overexpression renders the cells more susceptible to ferroptosis (15). Hence, PUFAs must be in the membrane-bound form to be lethal when exposed to peroxidation. The cystine/glutamate reverse transporter (System Xc^-^) and GPX4 are effective suppressors of ferroptosis, while ACSL4 and LPCAT3 are identified as pro-ferroptosis due to their role in incorporating PUFAs into membrane lipids. Recent research has elucidated that ACSL4 is involved in a feedforward loop to regulate ferroptosis. Protein kinase C β type isoform 2 (PKCβII) is able to recognize the initial lipid peroxidation, promote the phosphorylation of ACSL4 on Thr328 to activate phospho-ACSL4, and facilitate the incorporation of PUFA into PLs, leading to the termination of the cells (9). ACSL4 is a signaling component that plays a role in regulating ferroptosis. E-cadherin is responsible for controlling the expression of ACSL4 through the Merlin–Hippo–Yap pathway, affecting the vulnerability of cells to ferroptosis (21). Moreover, other ACSL enzymes, such as α-eleostearic acid, require ACSL1 to exhibit pro-ferroptosis activity (22), while monounsaturated fatty acids require ACSL3 to perform anti-ferroptosis activities (23). The integration of fatty acids into membrane-bound lipids is believed to be vital for ACSL-dependent actions to modulate ferroptosis and convert fatty acids into CoA esters.

**Figure 1:**
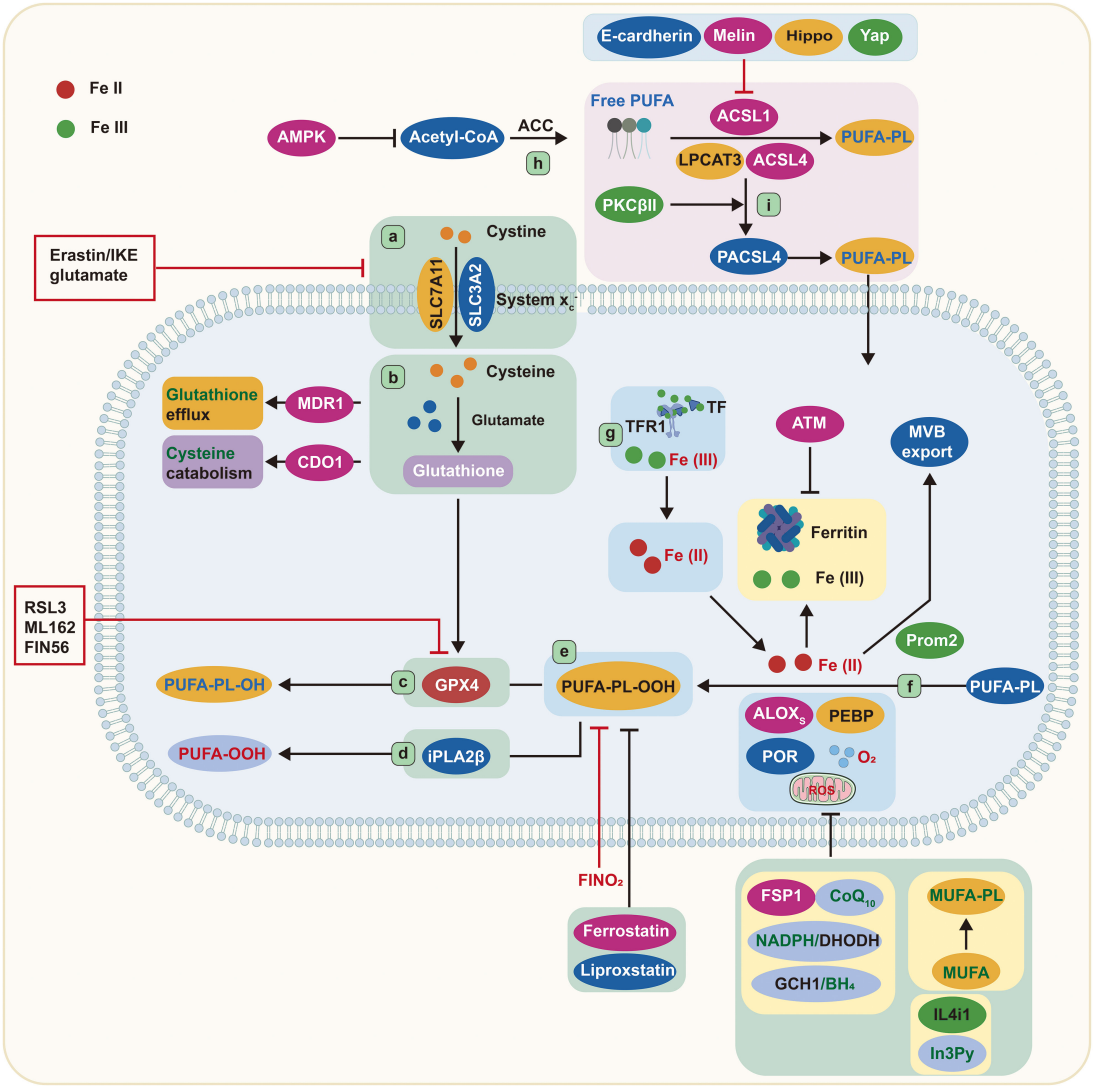
Core mechanisms of ferroptosis. Cystine is imported by System Xc^−^ and converted to the amino acid cysteine in the cell. Reduced glutathione is made from cysteine and glutamate and then used by GPX4 to convert reactive PUFA phospholipid hydroperoxides (PUFA-PL-OOH) to non-reactive and non-lethal PUFA phospholipid alcohols (PUFA-PL-OH). Alternatively, iPLA2β can eliminate the oxidized PUFAOOH tail from a phospholipid, preventing cell death. ALOXs oxidize PUFA-PLs in conjunction with PEBP1 and POR. TFR1 transports Fe (III) into cells, where it is reduced to Fe (II). Iron is stored in ferritin as Fe (III), where it is inaccessible and cannot induce ferroptosis. Iron export in MVBs is promoted by Prom2, which prevents ferroptosis. ACSL4, LPCAT3, and ACSL1 are all involved in the formation of PUFA-PLs from acetyl-CoA, and ACSL4 can be stimulated further by PKCβII phosphorylation. ALOXs, lipoxygenases; AMPK, adenosine-monophosphate-activated protein kinase; ACC, acetyl-coenzyme A carboxylase; ACSL, acyl-CoA synthetase long-chain family member; ATM, ATM serine/threonine kinase; BH4, tetrahydrobiopterin; CDO1, cysteine dioxygenase 1; CoA, coenzyme A; CoQ10, coenzyme Q10; cys, cysteine; DHODH, dihydroorotate dehydrogenase; FIN56, ferroptosis-inducer-56; FINO2, 1,2-dioxolane; FSP1, ferroptosis suppressor protein 1/AIFM2; GCH1, GTP cyclohydrolase 1; Glu, glutamate; GPX4, glutathione peroxidase 4; GSH, glutathione; IL4i1, interleukin-4-induced-1; In3Py, indole-3-pyruvate; iPLA2β, phospholipase A2 group VI; LPCAT3, lysophosphatidylcholine acyltransferase 3; lysoPL, lysophospholipid; MDR1, multiple drug resistance 1; MUFA, monounsaturated fatty acid; MVB, multivesicular body; NADPH, reduced nicotinamide adenine dinucleotide phosphate; PEBP1, phosphatidylethanolamine binding protein 1; PKCβII, protein kinase C β type isoform 2; PL, phospholipid; POR, cytochrome p450 oxidoreductase; Prom2, prominin-2; PUFA, polyunsaturated fatty acid; PUFA-PL-OOH, phospholipid with peroxidized polyunsaturated fatty acyl tail; ROS, reactive oxygen species; System Xc^−^, sodium-independent, anionic amino acid transport system; TF, transferrin; TFR1, transferrin receptor protein 1; Yap, Yes-associated protein.

According to recent findings, membrane lipids are key regulators of ferroptosis. The phospholipase A2 group VI (iPLA2β) can reduce p53-mediated ferroptosis by removing oxidized PUFA tails from PLs (24) (**Figure 1**), suggesting that oxidized PUFA must be present in a membrane-bound PL in order to trigger ferroptosis. The oxidation of PUFA tails has no effect on cell death once they are separated from PLs, implying that PUFA oxidation is intrinsically nontoxic. When oxidized PUFA-carrying lipids accumulate in cell membranes, the ferroptosis process is initiated (25, 26). Therefore, ferroptosis is better described as an accumulative process of lethal membrane-localized lipid peroxides instead of a type of oxidative stress.

It has been documented that one fatty acyl tail of PLs is mainly derived from a saturated fatty acid, and the other is from unsaturated fatty acids. Both fatty acyl tails are integral to ferroptosis (27). Nevertheless, uncommon PLs with two PUFA tails are also of great importance. Ether lipids such as plasmalogens and 2-arachidonoyl glycerol, which contain PUFAs, are associated with ferroptosis sensitivity (27). Thus, a variety of lipids with PUFA tails are involved in the induction of ferroptosis. Furthermore, energy stress can activate adenosine-monophosphate-activated protein kinase (AMPK), which, in turn, controls acetyl-CoA carboxylase, ultimately limiting PUFA production and reducing the susceptibility to ferroptosis (28) (**Figure 1**). Collectively, PUFA-containing membrane lipids, especially PLs and ether lipids, are important elements in ferroptosis.

### 2.2 Oxidative stress biology

GSH is a water-soluble tripeptide composed of glutamate, cysteine, and glycine amino acid residues. A deficiency in GSH impairs the cells’ ability to oppose free radicals and act as an antioxidant. GPX4 relies on GSH to decrease lipid peroxide. As a critical regulator of ferroptosis, GPX4 is the most potent anti-lipid peroxidase in the cell (29, 30). In order to produce GSH, System Xc^−^ is required to exchange extracellular cystine and intracellular glutamate. System Xc^−^ is composed of the solute carrier family 7 member 11 (SLC7A11) and the solute carrier family 3 member 2 (SLC3A2). By blocking System Xc^−^ or impeding the glutamate metabolic pathway, more ROS-induced lipid peroxidation and ferroptosis are triggered (31–33).

Erastin and RSL3 are two well-known compounds that induce ferroptosis (8). These compounds collaborate to prevent cystine absorption *via* System Xc^−^ and to inhibit GPX4, respectively (**Figure 1**). Moreover, other approaches aimed at depleting GSH can also render cells prone to ferroptosis. For example, MDR1 gene augments ferroptosis sensitivity by promoting GSH efflux, while cysteine dioxygenase 1 (CDO1) increases ferroptosis sensitivity by depleting cysteine and GSH (34, 35). Glutamate-cysteine ligase protects cells from ferroptosis by decreasing glutamate levels, converting them to γ-glutamyl peptides, and boosting GSH production (36) (**Figure 1**). Researchers have also discovered a compound, ferroptosis-inducer-56 (FIN56), that can degrade GPX4 and deplete coenzyme Q10 (CoQ10) *via* the mevalonate pathway, thus initiating ferroptosis (37). Additionally, GPX4 degradation *via* chaperone-mediated autophagy is another approach to induce ferroptosis (38) (**Figure 1**).

It is widely accepted that GPX4 plays an inhibitory role in ferroptosis; however, three GPX4-independent pathways have been determined. These include ferroptosis suppressor protein 1(FSP1)/CoQ10, GTP cyclohydrolase1 (GCH1)/tetrahydrobiopterin (BH4), and dihydroorotate dehydrogenase (DHODH) (**Figure 1**). GPX4 is the primary endogenous defense mechanism against lipid peroxidation, while CoQ10 provides secondary protection against lipid peroxidation-induced ferroptosis. Research has indicated that FSP1 could be instrumental in reducing CoQ10 when nicotinamide adenine dinucleotide phosphate (NADPH) is present (39, 40). Furthermore, NADPH has been identified as an effective signal for gauging resistance to ferroptosis. The cytoplasmic phosphatase metazoan spot homologue 1 (MESH1) is able to affect the susceptibility to ferroptosis by altering the levels of NADPH (41, 42).

In the past 2 years, two novel pathways unrelated to GPX4 have been discovered to inhibit lipid peroxidation associated with ferroptosis. The first pathway involves GCH1, which was identified as a ferroptosis inhibitor through a CRISPR gain-of-function screen (27). GCH1 plays an essential role in preventing ferroptosis by synthesizing the lipophilic antioxidant BH4, which helps reduce lipid peroxidation, and by remodeling lipid membranes to increase the reduced CoQ10 while decreasing the PUFA-PLs. The second pathway involves DHODH, a mitochondrial suppressor of ferroptosis that downregulates the abundance of mitochondrial CoQ10 (43). Cells expressing high levels of GCH1 or DHODH possess a greater ability to withstand ferroptosis. Emerging evidence reveals that the amino acid oxidase interleukin (IL)-4-induced-1 (IL4i1) can produce indole-3-pyruvate (In3Py) to inhibit ferroptosis through radical scavenging and altering a gene expression profile (44) (**Figure 1**).

### 2.3 Iron homeostasis

The Fenton reaction, promoted by iron-dependent enzymes and labile iron pool, is responsible for the peroxidation of membrane-bound, PUFA-containing lipids (45, 46). 15-Lipoxygenase can form a complex by binding with phosphatidylethanolamine binding protein 1 (PEBP1), which alters the enzyme’s substrate selectivity from free PUFAs to the PUFA tails of PLs (47) (**Figure 1**). In addition, 12-lipoxygenase is necessary for p53-mediated ferroptosis (48). Cytochrome P450 oxidoreductase also participates in lipid peroxidation during ferroptosis (49), implying that multiple iron-containing enzymes can stimulate lipid peroxidation (**Figure 1**).

The availability of ferritin has a direct effect on the volume of the labile iron pool. Manipulating ferritin levels through ferritinophagy can influence the iron abundance and the susceptibility to ferroptosis (50, 51). Ataxia telangiectasia kinase (ATM) has been reported to control the susceptibility to ferroptosis by affecting ferritin levels (52). Other mechanisms that regulate the intracellular iron abundance, such as the exportation of iron *via* various multivesicular bodies and exosomes carrying ferroportin or prominin2-mediated ferritin, can also affect the cellular sensitivity to ferroptosis, as they deplete the cellular iron and accelerate the lipid peroxidation (53) (**Figure 1**).

Ferroptosis can be triggered by the interaction of endoperoxide molecules with iron. Abrams et al. identified 1,2-dioxolane (FINO2) as the fourth class of chemicals able to induce ferroptosis (54). Unlike other chemicals which cause ferroptosis, FINO2 oxidizes Fe (II) to Fe (III). Consequently, iron chelators are more effective in counteracting its effects than they are against other substances. Upon FINO2 treatment, cells exhibit a more extensive oxidized lipid profile, indicating that FINO2 carries out a Fenton reaction with Fe (II), creating alkoxyl radicals that lead to lipid peroxidation (55). Therefore, endoperoxides are regarded as the origin of pro-ferroptosis agents, which can act as intrinsic triggers of ferroptosis in certain conditions.

Cells maintain a labile iron pool of Fe (II), linked to low-molecular-weight compounds, such as GSH. A decline in GSH can cause Fe (II) to be employed for Fenton chemistry, leading to lipid peroxide accumulation and ferroptosis (56). The transportation of the GSH–iron complex to ferritin, mediated by chaperone poly(rC) binding protein 1, is necessary for iron storage in ferritin. As a result, a decrease in GSH can lead to a labile iron availability (57).

## 3 Ferroptosis in immune cells

**Figure 2:**
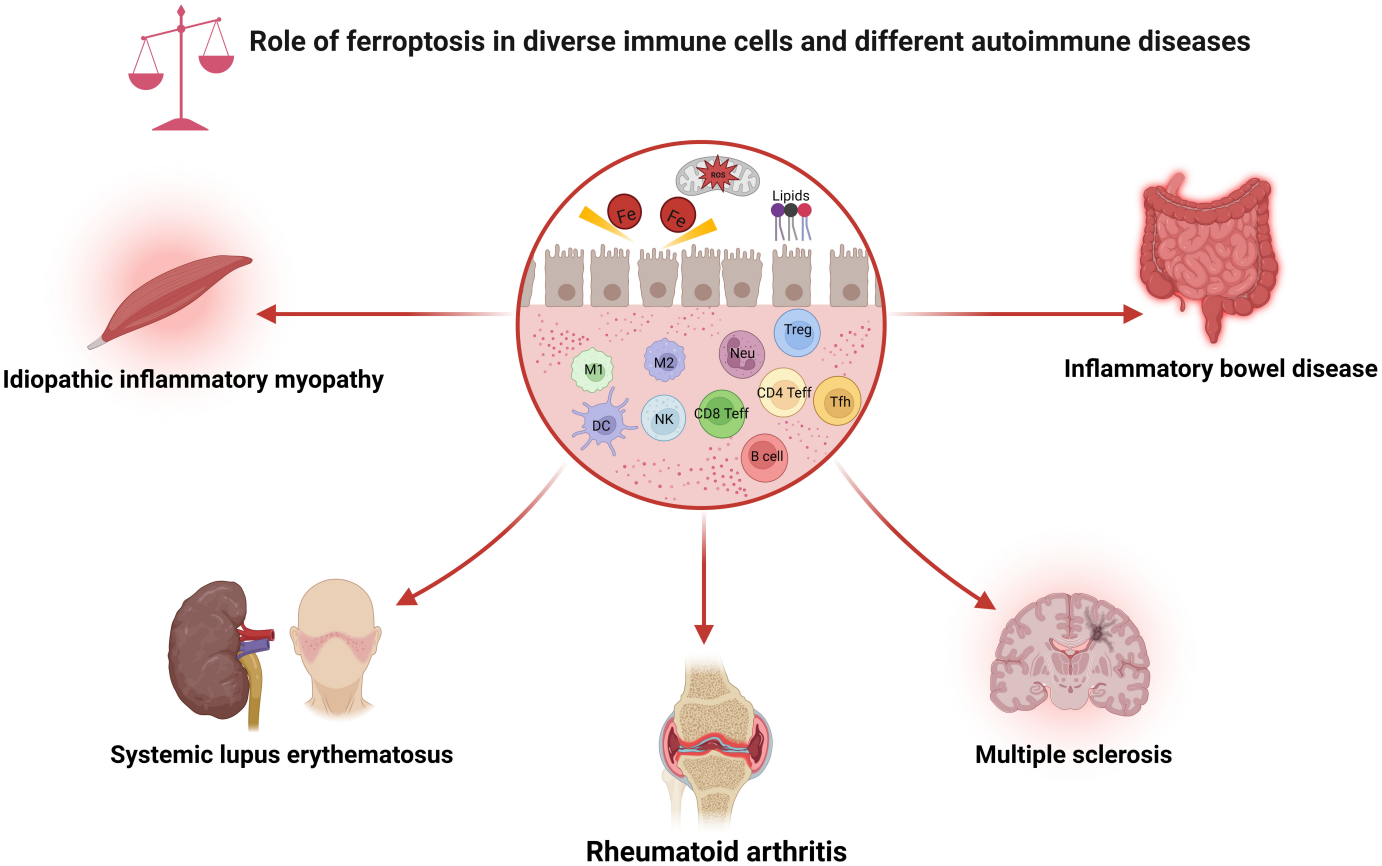
Role of ferroptosis in diverse immune cells and different autoimmune diseases. As shown, ferroptosis has been associated with various immune cell subpopulations and autoimmune diseases. CD4 Teff, CD4+ effector T cell; CD8 Teff, CD8+ effector T cell; DC, dendritic cell; M1, macrophage 1; M2, macrophage 2; NK, natural killer cell; Neu, neutrophils; ROS, reactive oxygen species; Tfh, follicular helper T cell; Treg, regulatory T cell.

The immune system is usually separated into two distinct categories: innate and adaptive immunity. Ferroptosis can influence both the quantity and function of the immune cells. Intriguingly, cells undergoing ferroptosis can be recognized by immune cells, subsequently promoting various immune responses. This section will explore how ferroptosis affects various types of immune cells, which may guide the development of tailored therapy (**Figure 2**).

### 3.1 Neutrophils

Neutrophil granulocytes are the first line of defense against inflammation in tissues, utilizing phagocytosis, degranulation, and generation of neutrophil extracellular traps (NETs) to combat pathogens. Peptidyl arginine deiminase 4 (PADI4) and NADPH oxidase are necessary for NET synthesis (58, 59). Sulfasalazine has been found to increase the nonenzymatic synthesis of oxidized PLs, resulting in the formation of NETs in neutrophils, which is mediated by lipid peroxidation (60). Furthermore, neutrophils are able to cause lipid peroxidation in glioblastoma cells by incorporating myeloperoxidase (61).

Following injury, the aggregation of neutrophils significantly contributes to activating and maintaining early inflammatory responses, which are necessary for the initial stage of the healing process and introduction of further tissue damage (62). In animal studies, the stimulation of ferroptosis accelerates the neutrophil migration to the myocardium following heart transplantation, resulting in cardiac injury (63). In contrast, Fer-1, a specific ferroptosis inhibitor, has been shown to reduce the lipid peroxidation end products, thus inhibiting cardiomyocyte death and preventing neutrophil recruitment after heart transplantation (63). Arachidonate lipoxygenase 15 (ALOX15), a member of the ALOX family, has the potential to facilitate the production of lipid peroxidation (64). Accordingly, ALOX15 can serve as a direct target of Fer-1. Considering that Fer-1 suppresses the sterile cardiac inflammation caused by ferroptosis, future exploration is anticipated to ascertain whether ALOX15 deletion can also protect against cardiac injury.

### 3.2 Natural killer cells

Natural killer (NK) cells possess the ability to destroy virus-infected and tumor cells without prior sensitization and are proficient interferon-γ (IFN-γ) producers (65). Kong et al. found that IFN-γ can accelerate GSH consumption, increase lipid peroxidation, and render hepatocellular carcinoma more vulnerable to ferroptosis by promoting Janus kinase/signal transducer and activator of transcription (JAK/STAT) signaling while suppressing System Xc^−^ (66). However, in the tumor microenvironment (TME), NK cells become impaired, which may be linked to ferroptosis. In NK cells, exposure to TME downregulates the expression of CD71, CD98, and glucose transporter type 1 (GLUT-1) but elevates the abundance of proteins related to ferroptosis, oxidative damage, and lipid peroxidation (67). To investigate the role of ferroptosis enhancers or suppressors in tumor-associated NK cells, more intensive research is warranted in the future.

### 3.3 Dendritic cells

Dendritic cells (DCs) are a heterogeneous family of hematopoietic cells that are specialized for recognizing pathogens (68). DCs are involved in T-cell activation and the presentation of antigens. Under normal circumstances, DCs are immature, with a high capacity for capturing antigens, limited ability to produce cytokines, and low levels of co-stimulatory molecules (69). In the presence of infection or tissue damage, DCs enter a mature state characterized by the high expression of major histocompatibility complex (MHC) and co-stimulatory molecules, enhanced cytokine release, and increased cell motility. In the TME, DCs play a key role in capturing, processing, and presenting tumor-associated antigens, as well as providing co-stimulation and soluble factors to regulate T-cell responses (70). Upon encountering an antigen, immature DCs transform into mature DCs due to their robust antigen phagocytic capacities. DCs will interact with T cells and induce an immunological response (69, 71). While GPX4-induced inhibition of ferroptosis in T and B cells has been linked to weakened adaptive immune systems, the underlying molecular basis of GPX4 inhibition in DCs remains unknown.

Han et al. demonstrated that the GPX4 inhibitor RSL3 selectively promotes ferroptosis in DCs (72). This phenomenon results in DCs losing the capability to release MHC class I and several pro-inflammatory cytokines in response to the maturation of lipopolysaccharide (LPS) and failing to stimulate CD8+ T cells to produce IFN-γ. In DCs, peroxisome proliferator-activated receptor-γ (PPARγ), a nuclear receptor, has been shown to promote RSL3-caused ferroptosis. PPARγ knockout restores the maturation and function of DCs (72). Therefore, PPARγ-associated ferroptosis in DCs hinders anti-tumor immunity in mice, which suggests that ferroptotic DCs possess a novel function in fostering an immunosuppressive TME.

It is reported that sestrin2 (Sesn2) is a highly evolved, stress-responsive protein essential for oxidative stress resistance. Upregulation of Sesn2 was found to have antioxidative properties in inflammatory settings (73). However, the exact role of Sesn2 in ferroptosis related to the immune system is still unclear. In LPS-stimulated DCs, a recent study has found that activation of Sesn2 in the ferroptosis network can regulate the immunological function of DCs (74). Sesn2 expression also increases in response to LPS stimulation, but LPS treatment leads to ferroptosis and impedes DC maturation and motivation. Furthermore, deletion of Sesn2 intensifies LPS-induced ferroptosis and impairs the ferroptosis and immunological response of DCs after cecal ligation and puncture (74). These findings suggest that Sesn2 may act as a negative regulator of ferroptosis in the context of sepsis.

### 3.4 Macrophages

Macrophages are a pivotal type of immune cells in most organisms and are responsible for tissue homeostasis and defense. Studies have revealed that macrophages derived from human monocytes are less capable of phagocytosing during ferroptosis than their apoptotic counterparts in Jurkat T cells. This may be because the latter releases more molecules that signal macrophages to consume them (75). This observation supports the hypothesis that ferroptosis, a type of nonapoptotic cell death, has a greater possibility of inducing inflammation than apoptosis. Meanwhile, during the ferroptosis, cells release oxygenated phosphatidylethanolamines on the outer plasma membrane, which are detected by macrophages *via* toll-like receptor 2, allowing the clearance of ferroptosis cells (76).

Macrophages participate in iron regulation by retrieving and scavenging iron from aged erythrocytes, which may lead to macrophage ferroptosis and impair their immunological function (77). Research has shown that administration of red blood cells that have been affected by cold temperatures can induce erythrophagocytosis, which is related to the Fer-1-induced inhibition in splenic red pulp macrophages (78). Additionally, exogenous ferric citrate-induced cell death in bone marrow-derived macrophages (BMDM) may be prevented by Fer-1, underlining the involvement of iron in promoting ferroptosis in macrophages. Iron has been found to upregulate SLC7A11mRNA as a protective mechanism *via* increasing activities of the ROS-nuclear factor erythroid 2-related factor 2 (NRF2) signaling, which helps to inhibit ferroptosis in BMDM (79). Furthermore, transforming growth factor-β 1 (TGFβ1), released by macrophages, modulates the expression of SLC7A11 *via* the Smad pathway. In macrophages, silence of the SLC7A11 gene promotes iron overload-induced ferroptosis, suggesting that the TGFβ signaling pathway is crucial for regulating macrophage ferroptosis (21, 80, 81).

Interestingly, the susceptibility of macrophages to ferroptosis varies significantly between the subtypes (82). This is due to the fact that M1 macrophages, which are pro-inflammatory, possess higher concentrations of inducer nitric oxide synthase (iNOS), leading to a greater production of nitric oxide free radicals and stronger protection against lipid peroxidation, when compared to M2 macrophages, which are anti-inflammatory and have lower iNOS concentrations (83). Additionally, hypoxia-induced downregulation of nuclear receptor coactivator 4 and p53 polymorphisms also control the susceptibility of macrophages to ferroptosis (50, 51, 84). Therefore, it is essential to explore the transcriptional patterns in different populations of macrophages in various organs to gain a deeper understanding of their influence on ferroptosis sensitivity.

### 3.5 T lymphocytes

#### 3.5.1 Cytotoxic and helper T cells

Recent studies have suggested that both ferroptosis and lipid peroxidation modulate the function of CD8+cytotoxic and CD4+helper T cells. SLC7A11, which is not present in naive human CD4+ T cells, is substantially increased due to the activation of T cells (85, 86). The proliferation and activation of T cells are associated with a decrease in extracellular GSH levels that subsequently result in a reduction of intracellular GSH levels (87). Furthermore, downregulation of GPX4 *via* genetic deletion or chemical inhibition can lead to ferroptosis in T cells, while upregulation of GPX4 and genetic deletion of ACSL4 can suppress ferroptosis in CD8+ T cells (88, 89). Interestingly, ablation of GPX4 in T cells disrupts CD8+ T-cell homeostasis in the periphery but does not affect thymopoiesis in mice (88). GPX4-knockout CD8+ T cells and CD4+ T cells are unable to develop during acute infection, which can be reversed by the administration of vitamin E, a powerful lipid-soluble antioxidant (88). Ferroptosis in CD8+ T cells promotes the anti-tumor function through activating the JAK/STAT pathway, which limits cystine absorption by tumor cells (90). Notably, iron overload can cause inequalities in the proportion of CD4+ and CD8+ T cells, a rise of ROS levels in T cells, and DNA damage (91). Hence, regulating the interferon and iron metabolism in T cells is a possible direction for improving the efficacy of T cell-mediated immunotherapy (90, 92).

#### 3.5.2 Follicular helper T cells

CD4+ T cells have a specialized subset named follicular helper T (Tfh) cells associated with the germinal center (GC) response. The GC is where B cells differentiate into memory cells and long-lasting plasma cells. Within the context of autoimmunity, pathogenic autoantibodies are produced in response to abnormal Tfh cell response (93, 94). On the other hand, Tfh cells play a protective role in the humoral immunity stimulated by vaccination (95, 96). Recent research has highlighted the importance of ferroptosis in maintaining the function of Tfh cells generated by an acute viral infection or protein immunization (97–99). GPX4, a key lipid peroxidation scavenger, is critical for the survival of Tfh cells. To be specific, deletion of GPX4 in T cells resulted in loss of Tfh cells and GC responses in immunized mice, suggesting that Tfh cells are particularly susceptible to ferroptosis. Selenium supplementation could increase GPX4 expression in T cells, thereby leading to an increase in Tfh cell numbers and improved antibody responses in vaccinated mice and young adults post-influenza vaccination (97). These findings demonstrate the significance of the selenium–GPX4–ferroptosis pathway in maintaining Tfh cell homeostasis, which can be used to improve Tfh cell performance during infection and after vaccination.

Accumulating evidence has revealed that ferroptosis functions as a key factor in regulating Tfh cell activity in immunization mice models and controlling the preservation of viral-infected memory Tfh cells (98). In mice with acute lymphocytic choriomeningitis virus, CD4+ T cells with the silence of mechanistic target of rapamycin complex-2 (mTORC2) impede the formation and growth of memory CD4+ T cells, which is attributed to the IL-7-activated mTORC2–AKT–GSK3 signaling pathway, leading to excessive GPX4 peroxidase and suppression of ferroptosis in memory cells (98). Moreover, the mTORC2 signaling pathway can promote Tfh cell development in mice following viral/protein antigen vaccination (100–102). More research is necessary to investigate how the mTORC2 signaling in Tfh cells drives ferroptosis and cell lineage differentiation.

#### 3.5.3 Regulatory T cells

Regulatory T (Treg) cells are key components of immunological tolerance and anti-tumor immunity (103–105), yet the mechanisms underlying the related cellular redox equilibrium are still unknown. To elucidate the role of ferroptosis in Treg cells, GPX4 is selectively deleted, which leads to an impairment of immunological balance without affecting the longevity of Treg cells (106). When exposed to T-cell receptor and CD28 simultaneously, GPX4-deficient Treg cells experience augmented lipid peroxidation and ferroptosis. Restricting access to lipid peroxidation and limiting iron availability reverses ferroptosis in GPX4-knockout Treg cells. Furthermore, GPX4-knockout Treg cells show increased production of mitochondrial superoxide and IL-1β, which, in turn, amplify Th17 cellular responses. Lastly, specific ablation of GPX4 in Treg cells suppresses tumor growth and boosts anti-tumor immunity (106). These findings actively support the importance of GPX4 in preventing activated Treg cells from ferroptosis and lipid peroxidation and suggest a potential therapeutic avenue to optimize cancer therapy.

### 3.2 B cells

B cells can be divided into three distinct compartments: a long-lived pool that recycles through secondary lymphoid organs, a static compartment concentrated in the marginal zone B (MZB) cells in the splenic marginal zone, and B1 cells that mature in the peritoneal and pleural cavities (107). GPX4 has been identified as a critical factor for the development and function of MZB and B1 cells but does not have such effects on follicular B cells (108). In mice, deletion of GPX4 triggers ferroptosis and inhibits the production of IgM antibodies in response to *Streptococcus pneumoniae*. Furthermore, upregulation of CD36, a fatty acid transporter protein, has been observed in MZB and B1 cells, which can promote fatty acid absorption and ferroptosis sensitivity (108). These findings shed light on the mechanism underlying the redox regulation that affects the B-cell homeostasis. Of note, B1a cells are more reliant on external fatty acid intake and mobilization *via* autophagy for cell self-renewal and survival than follicular B2 cells (109). This is because lipid droplets are the primary storage location for fatty acids, thus preventing PUFA oxidation (110). Autophagy-mediated lipid droplet destruction (lipophagy) causes ferroptosis by increasing the intracellular free fatty acids (111). This suggests that lipophagy may influence B-cell activity *via* ferroptosis, a hypothesis that requires further validation. Moreover, the ferroptosis inducer erastin downregulates the activity of bone morphogenetic protein (BMP) family members and promotes the differentiation and growth of human peripheral blood mononuclear cells into B and NK cells (112). BMPs are part of the TGFβ superfamily, which can regulate the cellular activities of B, NK, and myeloid cells (113). The above observations provide new insights into the role of lipid peroxidation in B-cell development and function.

Targeting ferroptosis constitutes a promising strategy for B cell-related complex diseases. For instance, through blocking the System Xc^−^ activity, erastin triggers ferroptosis, eventually inhibiting tumor growth in mouse models of lymphoma (114). Dimethyl fumarate decreases the levels of GSH and GPX4 and increases the expression of 5-lipoxygenase in GC B-cell diffuse large B-cell lymphoma, which helps induce ferroptosis by impeding nuclear factor-κB (NF-κB) and JAK/STAT signaling pathway (115). Furthermore, a protein–protein interaction network analysis has suggested that ferroptosis may be strongly correlated to B-cell receptor signaling and IgA synthesis in immune-deficient intestinal tissues (116).

## 4 Ferroptosis in autoimmune diseases

Great attention has been paid to the involvement of ferroptosis in various human disorders. Depending on the specific conditions, ferroptosis can be either pathogenic or therapeutic. Here, we will discuss the role of ferroptosis in different autoimmune diseases and highlight the emerging therapeutic opportunities and associated challenges with future prospects (**Figure 2**).

### 4.1 Ferroptosis and systemic lupus erythematosus

Systemic lupus erythematosus (SLE) is a prototype of autoimmune disease characterized by chronic inflammation, high level of autoantibody production, and multiple systems involvement (117). A recent study has revealed the immunopathogenic effects of neutrophil ferroptosis in SLE (118). Compared to healthy controls, SLE patients displayed a reduction of GPX4 in neutrophils. Intervention with SLE serum or interferon-α (IFN-α) reduced GPX4 expression and caused neutrophil death, while inhibition of IFN-α restored neutrophil survival. Similarly, administration of ferroptosis inhibitors also reduced the neutrophil death caused by SLE serum. Mechanistically, a conserved binding site for the transcriptional repressor cAMP-responsive element modulator α (CREMα) was found in the GPX4 promoter. Under the influence of SLE serum or IFN-α, neutrophils derived from healthy controls showed stronger binding of CREMα to the GPX4 promoter and more nuclear accumulation, leading to GPX4 suppression and elevated lipid peroxidation (118). These findings propose a paradigm that places neutrophil ferroptosis at the center of the pathogenesis of SLE. Therefore, targeting GPX4 transcription and neutrophil ferroptosis may have therapeutic benefits for SLE patients.

Cao et al. recently demonstrated that iron overload can lead to the proliferation of Tfh cells, the release of pro-inflammatory cytokines, and the formation of autoantibodies in lupus mice (119). When mice were fed with a high-iron diet, a more significant antigen-specific GC response and a greater number of Tfh cells were observed, indicating that iron could assist in Tfh cells’ differentiation. However, iron chelation suppressed the development of Tfh cells. From the mechanism, iron accumulation in Tfh cells was regulated by the microRNA-21/3-hydroxybutyrate dehydrogenase-2 axis, which also strengthened the Fe (II)-dependent TET enzyme activity and demethylation of the BCL6 gene (119). This study implies that maintaining iron homeostasis is essential for eliminating pathogenic Tfh cells, which may lead to a more effective treatment for SLE patients.

Lupus nephritis (LN) is a severe form of organ damage that develops in most patients within 5 years of being diagnosed with SLE. Tubulointerstitial damage is a pathological hallmark of the lupus kidney, and its associated inflammation is critical to diagnose and predict the prognosis of LN (120–122). Renal tubular epithelial cells (RTECs) are an important factor in this milieu and are responsible for inducing interstitial inflammation and kidney injury (123). It has been reported that iron deposition in LN leads to albuminuria (124) and that RTECs are responsible for reabsorbing most of the filtered iron. These cells also undergo ferroptosis under pathological contexts (125–127). Consequently, it is plausible that iron accumulation in RTECs may aggravate inflammatory responses by increasing ROS production, ultimately resulting in renal failure.

### 4.2 Ferroptosis and rheumatoid arthritis

Rheumatoid arthritis (RA) is the typical autoimmune disease that primarily targets the synovial membrane of the joints, resulting in the synovium transforming into a hyperplastic and invasive tissue that destroys cartilage and bone (128). Although the exact mechanism of RA remains elusive, immune cells and fibroblast-like synoviocytes (FLS) are thought to be involved in its progression (129, 130).

Synovium hyperplasia is a key factor in the development of RA, which contributes to cartilage degradation and invasive pannus formation (131). According to preliminary results, ferroptosis may be involved in balancing the synovium growth and death. Ling et al. found a decline of ferroptosis in RA synovium and RA-FLS, with the upregulation of GPX4 and ferritin heavy chain 1 (FTH1) and the downregulation of ACSL4 (132). RNA sequencing (RNA-seq) and further verification showed that glycine modulates ferroptosis by inhibiting the expression of FTH1 and GPX4 and promoting the expression of S-adenosylmethionine in RA-FLS (132). Hence, these findings suggest that glycine supplementation can induce ferroptosis, which helps to reduce the RA-FLS proliferation and inflammation, consequently alleviating the progression of RA.

Building on the collagen-induced arthritis (CIA) mouse model, Wu et al. discovered that administration of imidazole ketone erastin (IKE), a ferroptosis inducer, could mitigate synovitis and prevent joint destruction (133). RNA-seq data further revealed two groups of FLS with different susceptibilities to IKE-induced ferroptosis. Notably, ferroptosis-resistant FLS exhibited a greater amount of tumor necrosis factor (TNF)-related transcriptome. Mechanistically, TNF facilitated cystine absorption and GSH production *via* activation of the NF-κB pathway to shield FLS from ferroptosis. Finally, combining low-dosage IKE with etanercept, a TNF inhibitor, triggered ferroptosis in FLS and delayed arthritis progression in the CIA model (133). Taken together, these results indicate that the combination of TNF inhibitor and ferroptosis inducer could be a promising therapy for RA treatment.

### 4.3 Ferroptosis and inflammatory bowel disease

Inflammatory bowel disease (IBD), including Crohn’s disease (CD) and ulcerative colitis (UC), is a chronic and progressive disorder with recurrent episodes (134, 135). Although the etiology of IBD remains unknown, it is believed that a cocktail of genetic factors, environment, intestinal microbiota, and immune responses are to blame (136). Recent studies have demonstrated the cell death’s role in the homeostasis of the intestinal epithelium in IBD (137). For example, Werner et al. discovered that iron supplementation could alter gut microbial homeostasis and exacerbate intestinal inflammation in a murine model of CD (138). Kobayashi et al. also found that a high-iron diet can increase the risk of UC. In contrast, a high intake of zinc could reduce mucosal ROS production and improve colonic symptoms in IBD (139). These findings reveal a potential crosstalk between IBD and ferroptosis, where the Fenton reaction causes oxidative stress by generating ROS from excess iron in the gut. Lipid peroxidation then occurs and leads to ferroptosis, which, in turn, causes the damage of intestinal epithelial cells (IECs) and destruction of the intestinal mucosal barrier. Conversely, ferroptosis inhibitors, such as Lip-1, Fer-1, and DFO, have been shown to improve disease symptoms and extend the diminution of the colon length in dextran sulfate sodium (DSS)-induced colitis in mice, indicating that suppression of ferroptosis has a positive effect on IBD (140–142). Intriguingly, a Western diet high in PUFAs can induce intestinal inflammation in mice lacking one allele of GPX4 in IECs, which implies that dietary PUFAs may be a trigger of GPX4-restricted mucosal inflammation phenocopying aspects of human CD (143).

NRF2 can regulate cellular antioxidant response by affecting the expression of genes associated with redox homeostasis. Mutations in the NRF2–lipid peroxidation–ferroptosis pathway have been observed in malignancies (144). Heme oxygenase-1 (HO-1), a cytoprotective enzyme, possesses anti-inflammatory properties and is involved in ferroptosis (145). Chen et al. determined that Fer-1 could alleviate the symptoms of DSS-induced colitis through the NRF2/HO-1 signaling pathway, suggesting that the NRF2 pathway may be a key factor mediating the ferroptosis in UC (140). ER stress has also been linked to ferroptosis in the pathogenesis of IBD. Xu et al. demonstrated that ferroptosis contributes to UC *via* ER stress-mediated IECs death; on the other hand, the phosphorylation of NF-κB-p65 can inhibit ER stress-mediated IEC ferroptosis to mitigate UC (141). Collectively, ferroptosis inhibition may be expected to open a new avenue for IBD treatment.

### 4.4 Ferroptosis and multiple sclerosis

Multiple sclerosis (MS), a chronic inflammatory condition of the central nervous system, is characterized by neuroinflammation, demyelination, oligodendrocyte loss, and neurodegeneration (146, 147). Owing to their capacity to modify the transcriptional profile and prompt multiple inflammatory phenotypes, microglia are of great significance in the development of MS (148).

Hu et al. revealed that the gray matter of MS patients and the spinal cord of experimental autoimmune encephalomyelitis mice both display decreased expression of GPX4 (149). This pioneering investigation uncovered the correlation between ferroptosis, microglia-driven neuroinflammation, and MS. Research has shown that cuprizone, a commonly used copper chelator, exacerbates the rapid loss of oligodendrocytes and demyelination caused by ferroptosis (150). *In vitro*, ferroptosis activates inflammatory responses in microglia, and the systemic inflammation caused by LPS stimulation can be partially inhibited by Fer-1 (151). Interestingly, recent studies have demonstrated that microglia are resistant to ferroptosis (83, 150). Compared to anti-inflammatory counterparts, pro-inflammatory microglia performed increased resistance to ferroptosis, likely because of an elevated NRF2 expression and a greater abundance of iNOS/nitric oxide. Furthermore, pro-inflammatory microglia have been reported to promote distant inhibition of ferroptosis (83, 151), indicating that these cells possess the anti-ferroptosis properties in neuroinflammation. However, the precise mechanism of ferroptosis in MS is yet to be elucidated.

### 4.5 Ferroptosis and idiopathic inflammatory myopathy

Idiopathic inflammatory myopathy (IIM), also known as myositis, is a heterogeneous group of autoimmune disorders that often involve persistent inflammation of muscle tissue, with various clinical presentations and different prognoses (152). It has been proposed that the pathophysiology of IIM could be attributed to increased cell death, inadequate removal of cell debris, and excessive autoantigens and pro-inflammatory cytokines, which eventually result in over-stimulated immunological and inflammatory responses (152). Hyperferritinemia is a feature identified in IIM-related interstitial lung disease that has been linked to disease severity and prognosis (153–156). Moreover, the mitochondrial abnormalities and elevated ROS levels have also been implicated in the pathogenesis of IIM (157–159). Therefore, it is plausible that ferroptosis may be involved in the occurrence and development of IIM, and further research is required to fully understand the inherent connection.

## 5 Conclusion and perspectives

With the growing importance of ferroptosis in various autoimmune disorders, it is emerging as a promising therapeutic target. To better understand its role, more intensive studies must be conducted to explore how it affects the different cells, tissues, and the entire organism.

Considerable progress has been made toward developing new drugs for ferroptosis, utilizing the well-known targets of the pathological conditions (160, 161). High-throughput screening and automation, as well as artificial intelligence-based strategies, can be used to identify novel ferroptosis modulators and the underlying mechanisms. Additionally, illuminating the relationship between structure and function and optimizing small molecules can expedite the production of new therapeutic agents. Finally, pharmacological research is needed to ensure that drugs are delivered to the necessary organs, such as the inflammatory joints of RA, with optimum safety and efficiency in a wide range of autoimmune diseases.

Research has made great strides in comprehending the role of ferroptosis in autoimmune diseases. However, a few important questions remain to be answered for the effective clinical application of ferroptosis-focused treatments. To begin with, can biomarkers of ferroptosis in autoimmune diseases be accurately identified with precision and sensitivity? Moreover, which immunological reactions or phases of the disease should be the target of ferroptosis? It is still unclear how, where, and when these culminate in cell death. Furthermore, there is an urgent need to develop innovative methods and elucidate mechanisms for selectively controlling ferroptosis in a way that does not harm healthy cells while treating autoimmune diseases. Addressing these questions could provide useful new insights into ferroptosis and broaden its application to various biological fields.

## Author contributions

JF conceived and wrote the manuscript. TJ collected the references. DH supervised the study and revised the manuscript. All authors have approved the submitted version.

## Glossary

**Table:**

ACSL	acyl-CoA synthetase long-chain family
ALOX15	arachidonate lipoxygenase 15
AMPK	adenosine-monophosphate-activated protein kinase
ATM	ATM serine/threonine kinase
BH4	tetrahydrobiopterin
BMDM	bone marrow-derived macrophages
BMP	bone morphogenetic protein
CD	Crohn’s disease
CDO1	cysteine dioxygenase 1
CIA	collagen-induced arthritis
CoA	coenzyme A
CoQ10	coenzyme Q10
CREMα	cAMP-responsive element modulator α
DC	dendritic cell
DFO	deferoxamine
DHODH	dihydroorotate dehydrogenase
DSS	dextran sulfate sodium
ER	endoplasmic reticulum
Fer-1	ferrostatin-1
FIN56	ferroptosis-inducer-56
FINO2	1,2-dioxolane
FLS	fibroblast-like synoviocytes
FSP-1	ferroptosis suppressor protein 1
FTH1	ferritin heavy chain 1
GC	germinal center
GCH1	GTP cyclohydrolase1
GLUT-1	glucose transporter type 1
GPX4	glutathione peroxidase 4
GSH	glutathione
HO-1	heme oxygenase-1
IBD	inflammatory bowel disease
IEC	intestinal epithelial cells
IFN-α	interferon-α
IFN-γ	interferon-γ
IIM	idiopathic inflammatory myopathy
IKE	imidazole ketone erastin
IL	interleukin
iNOS	inducer nitric oxide synthase
iPLA2β	phospholipase A2 group VI
JAK/STAT	Janus kinase/signal transducer and activator of transcription
Lip-1	liproxstatin-1
Lipophagy	autophagy-mediated lipid droplet destruction
LN	lupus nephritis
LPCAT	lysophatidylcholine acyltransferases
LPS	lipopolysaccharide
MESH1	metazoan spot homologue 1
MHC	major histocompatibility complex
MS	multiple sclerosis
mTORC2	mechanistic target of rapamycin complex-2
MZB	marginal zone B
NADPH	nicotinamide adenine dinucleotide phosphate
NETs	neutrophil extracellular traps
NF-κB	nuclear factor-κB
NK	natural killer
NRF2	nuclear factor erythrid-2 related factor 2
PADI4	peptidyl arginine deiminase 4
PC	phosphatidylcholine
PEBP1	phosphatidylethanolamine binding protein 1
PL	phospholipids
PKCβII	protein kinase C β
PPARγ	peroxisome proliferator-activated receptor-γ
PUFA	polyunsaturated fatty acid
RA	rheumatoid arthritis
RNA-seq	RNA sequencing
ROS	reactive oxygen species
RSL-3	RAS-selective lethal 3
RTEC	renal tubular epithelial cells
Sesn2	sestrin 2
SLC3A2	solute carrier family 3 member 2
SLC7A11	solute carrier family 7 member 11
SLE	systemic lupus erythematosus
System Xc^−^	cystine/glutamate reverse transporter
Tfh	follicular helper T cell
TGFβ1	transforming growth factor-β
TME	tumor microenvironment
TNF	tumor necrosis factor
Treg	regulatory T cell
UC	ulcerative colitis.

## References

[1] Yatim N Cullen S Albert ML. Dying cells actively regulate adaptive immune responses. Nat Rev Immunol (2017) 17(4):262–75. doi: 10.1038/nri.2017.9 28287107

[2] Dixon SJ Lemberg KM Lamprecht MR Skouta R Zaitsev EM Gleason CE. Ferroptosis: An iron-dependent form of nonapoptotic cell death. Cell (2012) 149(5):1060–72. doi: 10.1016/j.cell.2012.03.042 PMC3367386 22632970

[3] Lai B Wu CH Wu CY Luo SF Lai JH. Ferroptosis and autoimmune diseases. Front Immunol (2022) 13:916664. doi: 10.3389/fimmu.2022.916664 35720308 PMC9203688

[4] Stockwell BR Friedmann Angeli JP Bayir H Bush AI Conrad M Dixon SJ. Ferroptosis: A regulated cell death nexus linking metabolism, redox biology, and disease. Cell (2017) 171(2):273–85. doi: 10.1016/j.cell.2017.09.021 PMC5685180 28985560

[5] Tang D Chen X Kang R Kroemer G. Ferroptosis: Molecular mechanisms and health implications. Cell Res (2021) 31(2):107–25. doi: 10.1038/s41422-020-00441-1 PMC8026611 33268902

[6] Zheng J Conrad M. The metabolic underpinnings of ferroptosis. Cell Metab (2020) 32(6):920–37. doi: 10.1016/j.cmet.2020.10.011 33217331

[7] Dixon SJ Stockwell BR. The role of iron and reactive oxygen species in cell death. Nat Chem Biol (2014) 10(1):9–17. doi: 10.1038/nchembio.1416 24346035

[8] Xie Y Hou W Song X Yu Y Huang J Sun X. Ferroptosis: Process and function. Cell Death Differ (2016) 23(3):369–79. doi: 10.1038/cdd.2015.158 PMC5072448 26794443

[9] Zhang S Xin W Anderson GJ Li R Gao L Chen S. Double-edge sword roles of iron in driving energy production versus instigating ferroptosis. Cell Death Dis (2022) 13(1):40. doi: 10.1038/s41419-021-04490-1 35013137 PMC8748693

[10] Stockwell BR. Ferroptosis turns 10: Emerging mechanisms, physiological functions, and therapeutic applications. Cell (2022) 185(14):2401–21. doi: 10.1016/j.cell.2022.06.003 PMC9273022 35803244

[11] Harizi H Corcuff JB Gualde N. Arachidonic-Acid-Derived eicosanoids: Roles in biology and immunopathology. Trends Mol Med (2008) 14(10):461–9. doi: 10.1016/j.molmed.2008.08.005 18774339

[12] Shindou H Shimizu T. Acyl-Coa:Lysophospholipid acyltransferases. J Biol Chem (2009) 284(1):1–5. doi: 10.1074/jbc.R800046200 18718904

[13] Shindou H Hishikawa D Harayama T Yuki K Shimizu T. Recent progress on acyl coa: Lysophospholipid acyltransferase research. J Lipid Res (2009) 50 Suppl(Suppl):S46–51. doi: 10.1194/jlr.R800035-JLR200 PMC2674719 18931347

[14] Liao P Wang W Wang W Kryczek I Li X Bian Y. Cd8(+) T cells and fatty acids orchestrate tumor ferroptosis and immunity *Via* Acsl4. Cancer Cell (2022) 40(4):365–78.e6. doi: 10.1016/j.ccell.2022.02.003 35216678 PMC9007863

[15] Doll S Proneth B Tyurina YY Panzilius E Kobayashi S Ingold I. Acsl4 dictates ferroptosis sensitivity by shaping cellular lipid composition. Nat Chem Biol (2017) 13(1):91–8. doi: 10.1038/nchembio.2239 PMC5610546 27842070

[16] Kagan VE Mao G Qu F Angeli JP Doll S Croix CS. Oxidized arachidonic and adrenic pes navigate cells to ferroptosis. Nat Chem Biol (2017) 13(1):81–90. doi: 10.1038/nchembio.2238 27842066 PMC5506843

[17] Tanaka H Zaima N Sasaki T Yamamoto N Inuzuka K Yata T. Lysophosphatidylcholine acyltransferase-3 expression is associated with atherosclerosis progression. J Vasc Res (2017) 54(4):200–8. doi: 10.1159/000473879 28683445

[18] Hishikawa D Shindou H Kobayashi S Nakanishi H Taguchi R Shimizu T. Discovery of a lysophospholipid acyltransferase family essential for membrane asymmetry and diversity. Proc Natl Acad Sci U.S.A. (2008) 105(8):2830–5. doi: 10.1073/pnas.0712245105 PMC2268545 18287005

[19] Zhang Q Yao D Rao B Jian L Chen Y Hu K. The structural basis for the phospholipid remodeling by lysophosphatidylcholine acyltransferase 3. Nat Commun (2021) 12(1):6869. doi: 10.1038/s41467-021-27244-1 34824256 PMC8617236

[20] Dixon SJ Winter GE Musavi LS Lee ED Snijder B Rebsamen M. Human haploid cell genetics reveals roles for lipid metabolism genes in nonapoptotic cell death. ACS Chem Biol (2015) 10(7):1604–9. doi: 10.1021/acschembio.5b00245 PMC4509420 25965523

[21] Wu J Minikes AM Gao M Bian H Li Y Stockwell BR. Intercellular interaction dictates cancer cell ferroptosis *Via* Nf2-yap signalling. Nature (2019) 572(7769):402–6. doi: 10.1038/s41586-019-1426-6 PMC6697195 31341276

[22] Beatty A Singh T Tyurina YY Tyurin VA Samovich S Nicolas E. Ferroptotic cell death triggered by conjugated linolenic acids is mediated by Acsl1. Nat Commun (2021) 12(1):2244. doi: 10.1038/s41467-021-22471-y 33854057 PMC8046803

[23] Magtanong L Ko PJ To M Cao JY Forcina GC Tarangelo A. Exogenous monounsaturated fatty acids promote a ferroptosis-resistant cell state. Cell Chem Biol (2019) 26(3):420–32.e9. doi: 10.1016/j.chembiol.2018.11.016 30686757 PMC6430697

[24] Chen D Chu B Yang X Liu Z Jin Y Kon N. Ipla2beta-mediated lipid detoxification controls P53-driven ferroptosis independent of Gpx4. Nat Commun (2021) 12(1):3644. doi: 10.1038/s41467-021-23902-6 34131139 PMC8206155

[25] Sun WY Tyurin VA Mikulska-Ruminska K Shrivastava IH Anthonymuthu TS Zhai YJ. Phospholipase Ipla(2)Beta averts ferroptosis by eliminating a redox lipid death signal. Nat Chem Biol (2021) 17(4):465–76. doi: 10.1038/s41589-020-00734-x PMC8152680 33542532

[26] Beharier O Tyurin VA Goff JP Guerrero-Santoro J Kajiwara K Chu T. Pla2g6 guards placental trophoblasts against ferroptotic injury. Proc Natl Acad Sci U.S.A. (2020) 117(44):27319–28. doi: 10.1073/pnas.2009201117 PMC7959495 33087576

[27] Kraft VAN Bezjian CT Pfeiffer S Ringelstetter L Muller C Zandkarimi F. Gtp cyclohydrolase 1/Tetrahydrobiopterin counteract ferroptosis through lipid remodeling. ACS Cent Sci (2020) 6(1):41–53. doi: 10.1021/acscentsci.9b01063 31989025 PMC6978838

[28] Lee H Zandkarimi F Zhang Y Meena JK Kim J Zhuang L. Energy-Stress-Mediated ampk activation inhibits ferroptosis. Nat Cell Biol (2020) 22(2):225–34. doi: 10.1038/s41556-020-0461-8 PMC7008777 32029897

[29] Miao Y Chen Y Xue F Liu K Zhu B Gao J. Contribution of ferroptosis and Gpx4's dual functions to osteoarthritis progression. EBioMedicine (2022) 76:103847. doi: 10.1016/j.ebiom.2022.103847 35101656 PMC8822178

[30] Long L Guo H Chen X Liu Y Wang R Zheng X. Advancement in understanding the role of ferroptosis in rheumatoid arthritis. Front Physiol (2022) 13:1036515. doi: 10.3389/fphys.2022.1036515 36267583 PMC9576928

[31] Imai H Matsuoka M Kumagai T Sakamoto T Koumura T. Lipid peroxidation-dependent cell death regulated by Gpx4 and ferroptosis. Curr Top Microbiol Immunol (2017) 403:143–70. doi: 10.1007/82_2016_508 28204974

[32] Galluzzi L Bravo-San Pedro JM Vitale I Aaronson SA Abrams JM Adam D. Essential versus accessory aspects of cell death: Recommendations of the nccd 2015. Cell Death Differ (2015) 22(1):58–73. doi: 10.1038/cdd.2014.137 25236395 PMC4262782

[33] Seibt TM Proneth B Conrad M. Role of Gpx4 in ferroptosis and its pharmacological implication. Free Radic Biol Med (2019) 133:144–52. doi: 10.1016/j.freeradbiomed.2018.09.014 30219704

[34] Cao JY Poddar A Magtanong L Lumb JH Mileur TR Reid MA. A genome-wide haploid genetic screen identifies regulators of glutathione abundance and ferroptosis sensitivity. Cell Rep (2019) 26(6):1544–56.e8. doi: 10.1016/j.celrep.2019.01.043 30726737 PMC6424331

[35] Hao S Yu J He W Huang Q Zhao Y Liang B. Cysteine dioxygenase 1 mediates erastin-induced ferroptosis in human gastric cancer cells. Neoplasia (2017) 19(12):1022–32. doi: 10.1016/j.neo.2017.10.005 PMC5686465 29144989

[36] Kang YP Mockabee-Macias A Jiang C Falzone A Prieto-Farigua N Stone E. Non-canonical glutamate-cysteine ligase activity protects against ferroptosis. Cell Metab (2021) 33(1):174–89.e7. doi: 10.1016/j.cmet.2020.12.007 33357455 PMC7839835

[37] Shimada K Skouta R Kaplan A Yang WS Hayano M Dixon SJ. Global survey of cell death mechanisms reveals metabolic regulation of ferroptosis. Nat Chem Biol (2016) 12(7):497–503. doi: 10.1038/nchembio.2079 27159577 PMC4920070

[38] Wu Z Geng Y Lu X Shi Y Wu G Zhang M. Chaperone-mediated autophagy is involved in the execution of ferroptosis. Proc Natl Acad Sci U.S.A. (2019) 116(8):2996–3005. doi: 10.1073/pnas.1819728116 30718432 PMC6386716

[39] Bersuker K Hendricks JM Li Z Magtanong L Ford B Tang PH. The coq oxidoreductase Fsp1 acts parallel to Gpx4 to inhibit ferroptosis. Nature (2019) 575(7784):688–92. doi: 10.1038/s41586-019-1705-2 PMC6883167 31634900

[40] Doll S Freitas FP Shah R Aldrovandi M da Silva MC Ingold I. Fsp1 is a glutathione-independent ferroptosis suppressor. Nature (2019) 575(7784):693–8. doi: 10.1038/s41586-019-1707-0 31634899

[41] Shimada K Hayano M Pagano NC Stockwell BR. Cell-line selectivity improves the predictive power of pharmacogenomic analyses and helps identify nadph as biomarker for ferroptosis sensitivity. Cell Chem Biol (2016) 23(2):225–35. doi: 10.1016/j.chembiol.2015.11.016 PMC4792701 26853626

[42] Ding CC Rose J Sun T Wu J Chen PH Lin CC. Mesh1 is a cytosolic nadph phosphatase that regulates ferroptosis. Nat Metab (2020) 2(3):270–7. doi: 10.1038/s42255-020-0181-1 PMC7252213 32462112

[43] Mao C Liu X Zhang Y Lei G Yan Y Lee H. Dhodh-mediated ferroptosis defence is a targetable vulnerability in cancer. Nature (2021) 593(7860):586–90. doi: 10.1038/s41586-021-03539-7 PMC8895686 33981038

[44] Zeitler L Fiore A Meyer C Russier M Zanella G Suppmann S. Anti-ferroptotic mechanism of Il4i1-mediated amino acid metabolism. Elife (2021) 10:e64806. doi: 10.7554/eLife.64806 PMC7946422 33646117

[45] Shah R Shchepinov MS Pratt DA. Resolving the role of lipoxygenases in the initiation and execution of ferroptosis. ACS Cent Sci (2018) 4(3):387–96. doi: 10.1021/acscentsci.7b00589 PMC5879472 29632885

[46] Yang WS Kim KJ Gaschler MM Patel M Shchepinov MS Stockwell BR. Peroxidation of polyunsaturated fatty acids by lipoxygenases drives ferroptosis. Proc Natl Acad Sci U.S.A. (2016) 113(34):E4966–75. doi: 10.1073/pnas.1603244113 PMC5003261 27506793

[47] Wenzel SE Tyurina YY Zhao J St Croix CM Dar HH Mao G. Pebp1 wardens ferroptosis by enabling lipoxygenase generation of lipid death signals. Cell (2017) 171(3):628–41.e26. doi: 10.1016/j.cell.2017.09.044 29053969 PMC5683852

[48] Chu B Kon N Chen D Li T Liu T Jiang L. Alox12 is required for P53-mediated tumour suppression through a distinct ferroptosis pathway. Nat Cell Biol (2019) 21(5):579–91. doi: 10.1038/s41556-019-0305-6 PMC6624840 30962574

[49] Zou Y Li H Graham ET Deik AA Eaton JK Wang W. Cytochrome P450 oxidoreductase contributes to phospholipid peroxidation in ferroptosis. Nat Chem Biol (2020) 16(3):302–9. doi: 10.1038/s41589-020-0472-6 PMC7353921 32080622

[50] Gao M Monian P Pan Q Zhang W Xiang J Jiang X. Ferroptosis is an autophagic cell death process. Cell Res (2016) 26(9):1021–32. doi: 10.1038/cr.2016.95 PMC5034113 27514700

[51] Hou W Xie Y Song X Sun X Lotze MT Zeh HJ 3rd. Autophagy promotes ferroptosis by degradation of ferritin. Autophagy (2016) 12(8):1425–8. doi: 10.1080/15548627.2016.1187366 PMC4968231 27245739

[52] Chen PH Wu J Ding CC Lin CC Pan S Bossa N. Kinome screen of ferroptosis reveals a novel role of atm in regulating iron metabolism. Cell Death Differ (2020) 27(3):1008–22. doi: 10.1038/s41418-019-0393-7 PMC7206124 31320750

[53] Brown CW Amante JJ Chhoy P Elaimy AL Liu H Zhu LJ. Prominin2 drives ferroptosis resistance by stimulating iron export. Dev Cell (2019) 51(5):575–86.e4. doi: 10.1016/j.devcel.2019.10.007 31735663 PMC8316835

[54] Abrams RP Carroll WL Woerpel KA. Five-membered ring peroxide selectively initiates ferroptosis in cancer cells. ACS Chem Biol (2016) 11(5):1305–12. doi: 10.1021/acschembio.5b00900 PMC5507670 26797166

[55] Schilstra MJ Veldink GA Vliegenthart JF. The dioxygenation rate in lipoxygenase catalysis is determined by the amount of iron (Iii) lipoxygenase in solution. Biochemistry (1994) 33(13):3974–9. doi: 10.1021/bi00179a025 8142401

[56] Patel SJ Frey AG Palenchar DJ Achar S Bullough KZ Vashisht A. A Pcbp1-Bola2 chaperone complex delivers iron for cytosolic [2fe-2s] cluster assembly. Nat Chem Biol (2019) 15(9):872–81. doi: 10.1038/s41589-019-0330-6 PMC6702080 31406370

[57] Patel SJ Protchenko O Shakoury-Elizeh M Baratz E Jadhav S Philpott CC. The iron chaperone and nucleic acid-binding activities of Poly(Rc)-binding protein 1 are separable and independently essential. Proc Natl Acad Sci U.S.A. (2021) 118(25):e2104666118. doi: 10.1073/pnas.2104666118 34161287 PMC8237648

[58] Bianchi M Hakkim A Brinkmann V Siler U Seger RA Zychlinsky A. Restoration of net formation by gene therapy in cgd controls aspergillosis. Blood (2009) 114(13):2619–22. doi: 10.1182/blood-2009-05-221606 PMC2756123 19541821

[59] Li P Li M Lindberg MR Kennett MJ Xiong N Wang Y. Pad4 is essential for antibacterial innate immunity mediated by neutrophil extracellular traps. J Exp Med (2010) 207(9):1853–62. doi: 10.1084/jem.20100239 PMC2931169 20733033

[60] Yotsumoto S Muroi Y Chiba T Ohmura R Yoneyama M Magarisawa M. Hyperoxidation of ether-linked phospholipids accelerates neutrophil extracellular trap formation. Sci Rep (2017) 7(1):16026. doi: 10.1038/s41598-017-15668-z 29167447 PMC5700140

[61] Yee PP Wei Y Kim SY Lu T Chih SY Lawson C. Neutrophil-induced ferroptosis promotes tumor necrosis in glioblastoma progression. Nat Commun (2020) 11(1):5424. doi: 10.1038/s41467-020-19193-y 33110073 PMC7591536

[62] Kovtun A Messerer DAC Scharffetter-Kochanek K Huber-Lang M Ignatius A. Neutrophils in tissue trauma of the skin, bone, and lung: Two sides of the same coin. J Immunol Res (2018) 2018:8173983. doi: 10.1155/2018/8173983 29850639 PMC5937416

[63] Li W Feng G Gauthier JM Lokshina I Higashikubo R Evans S. Ferroptotic cell death and Tlr4/Trif signaling initiate neutrophil recruitment after heart transplantation. J Clin Invest (2019) 129(6):2293–304. doi: 10.1172/JCI126428 PMC6546457 30830879

[64] Ou Y Wang SJ Li D Chu B Gu W. Activation of Sat1 engages polyamine metabolism with P53-mediated ferroptotic responses. Proc Natl Acad Sci U.S.A. (2016) 113(44):E6806–E12. doi: 10.1073/pnas.1607152113 PMC5098629 27698118

[65] O'Brien KL Finlay DK. Immunometabolism and natural killer cell responses. Nat Rev Immunol (2019) 19(5):282–90. doi: 10.1038/s41577-019-0139-2 30808985

[66] Kong R Wang N Han W Bao W Lu J. Ifngamma-mediated repression of system xc(-) drives vulnerability to induced ferroptosis in hepatocellular carcinoma cells. J Leukoc Biol (2021) 110(2):301–14. doi: 10.1002/JLB.3MA1220-815RRR 34318944

[67] Poznanski SM Singh K Ritchie TM Aguiar JA Fan IY Portillo AL. Metabolic flexibility determines human nk cell functional fate in the tumor microenvironment. Cell Metab (2021) 33(6):1205–20.e5. doi: 10.1016/j.cmet.2021.03.023 33852875

[68] Pearce EJ Everts B. Dendritic cell metabolism. Nat Rev Immunol (2015) 15(1):18–29. doi: 10.1038/nri3771 25534620 PMC4495583

[69] Waisman A Lukas D Clausen BE Yogev N. Dendritic cells as gatekeepers of tolerance. Semin Immunopathol (2017) 39(2):153–63. doi: 10.1007/s00281-016-0583-z 27456849

[70] Wculek SK Cueto FJ Mujal AM Melero I Krummel MF Sancho D. Dendritic cells in cancer immunology and immunotherapy. Nat Rev Immunol (2020) 20(1):7–24. doi: 10.1038/s41577-019-0210-z 31467405

[71] Nagy L Szanto A Szatmari I Szeles L. Nuclear hormone receptors enable macrophages and dendritic cells to sense their lipid environment and shape their immune response. Physiol Rev (2012) 92(2):739–89. doi: 10.1152/physrev.00004.2011 22535896

[72] Han L Bai L Qu C Dai E Liu J Kang R. Pparg-mediated ferroptosis in dendritic cells limits antitumor immunity. Biochem Biophys Res Commun (2021) 576:33–9. doi: 10.1016/j.bbrc.2021.08.082 34478917

[73] Wang LX Zhu XM Luo YN Wu Y Dong N Tong YL. Sestrin2 protects dendritic cells against endoplasmic reticulum stress-related apoptosis induced by high mobility group box-1 protein. Cell Death Dis (2020) 11(2):125. doi: 10.1038/s41419-020-2324-4 32071292 PMC7028717

[74] Li JY Ren C Wang LX Yao RQ Dong N Wu Y. Sestrin2 protects dendrite cells against ferroptosis induced by sepsis. Cell Death Dis (2021) 12(9):834. doi: 10.1038/s41419-021-04122-8 34482365 PMC8418614

[75] Kloditz K Fadeel B. Three cell deaths and a funeral: Macrophage clearance of cells undergoing distinct modes of cell death. Cell Death Discovery (2019) 5:65. doi: 10.1038/s41420-019-0146-x 30774993 PMC6368547

[76] Luo X Gong HB Gao HY Wu YP Sun WY Li ZQ. Oxygenated phosphatidylethanolamine navigates phagocytosis of ferroptotic cells by interacting with Tlr2. Cell Death Differ (2021) 28(6):1971–89. doi: 10.1038/s41418-020-00719-2 PMC8185102 33432112

[77] Theurl I Hilgendorf I Nairz M Tymoszuk P Haschka D Asshoff M. On-demand erythrocyte disposal and iron recycling requires transient macrophages in the liver. Nat Med (2016) 22(8):945–51. doi: 10.1038/nm.4146 PMC4957133 27428900

[78] Youssef LA Rebbaa A Pampou S Weisberg SP Stockwell BR Hod EA. Increased erythrophagocytosis induces ferroptosis in red pulp macrophages in a mouse model of transfusion. Blood (2018) 131(23):2581–93. doi: 10.1182/blood-2017-12-822619 PMC5992863 29666112

[79] Wang H An P Xie E Wu Q Fang X Gao H. Characterization of ferroptosis in murine models of hemochromatosis. Hepatology (2017) 66(2):449–65. doi: 10.1002/hep.29117 PMC5573904 28195347

[80] Viswanathan VS Ryan MJ Dhruv HD Gill S Eichhoff OM Seashore-Ludlow B. Dependency of a therapy-resistant state of cancer cells on a lipid peroxidase pathway. Nature (2017) 547(7664):453–7. doi: 10.1038/nature23007 PMC5667900 28678785

[81] Kim DH Kim WD Kim SK Moon DH Lee SJ. Tgf-Beta1-Mediated repression of Slc7a11 drives vulnerability to Gpx4 inhibition in hepatocellular carcinoma cells. Cell Death Dis (2020) 11(5):406. doi: 10.1038/s41419-020-2618-6 32471991 PMC7260246

[82] Murray PJ. Macrophage polarization. Annu Rev Physiol (2017) 79:541–66. doi: 10.1146/annurev-physiol-022516-034339 27813830

[83] Kapralov AA Yang Q Dar HH Tyurina YY Anthonymuthu TS Kim R. Redox lipid reprogramming commands susceptibility of macrophages and microglia to ferroptotic death. Nat Chem Biol (2020) 16(3):278–90. doi: 10.1038/s41589-019-0462-8 PMC7233108 32080625

[84] Singh KS Leu JI Barnoud T Vonteddu P Gnanapradeepan K Lin C. African-Centric Tp53 variant increases iron accumulation and bacterial pathogenesis but improves response to malaria toxin. Nat Commun (2020) 11(1):473. doi: 10.1038/s41467-019-14151-9 31980600 PMC6981190

[85] Garg SK Yan Z Vitvitsky V Banerjee R. Differential dependence on cysteine from transsulfuration versus transport during T cell activation. Antioxid Redox Signal (2011) 15(1):39–47. doi: 10.1089/ars.2010.3496 20673163 PMC3110100

[86] Levring TB Hansen AK Nielsen BL Kongsbak M von Essen MR Woetmann A. Activated human Cd4+ T cells express transporters for both cysteine and cystine. Sci Rep (2012) 2:266. doi: 10.1038/srep00266 22355778 PMC3278673

[87] Pruett SB Obiri N Kiel JL. Involvement and relative importance of at least two distinct mechanisms in the effects of 2-mercaptoethanol on murine lymphocytes in culture. J Cell Physiol (1989) 141(1):40–5. doi: 10.1002/jcp.1041410107 2777901

[88] Matsushita M Freigang S Schneider C Conrad M Bornkamm GW Kopf M. T Cell lipid peroxidation induces ferroptosis and prevents immunity to infection. J Exp Med (2015) 212(4):555–68. doi: 10.1084/jem.20140857 PMC4387287 25824823

[89] Drijvers JM Gillis JE Muijlwijk T Nguyen TH Gaudiano EF Harris IS. Pharmacologic screening identifies metabolic vulnerabilities of Cd8(+) T cells. Cancer Immunol Res (2021) 9(2):184–99. doi: 10.1158/2326-6066.CIR-20-0384 PMC7864883 33277233

[90] Wang W Green M Choi JE Gijon M Kennedy PD Johnson JK. Cd8(+) T cells regulate tumour ferroptosis during cancer immunotherapy. Nature (2019) 569(7755):270–4. doi: 10.1038/s41586-019-1170-y PMC6533917 31043744

[91] Shaw J Chakraborty A Nag A Chattopadyay A Dasgupta AK Bhattacharyya M. Intracellular iron overload leading to DNA damage of lymphocytes and immune dysfunction in thalassemia major patients. Eur J Haematol (2017) 99(5):399–408. doi: 10.1111/ejh.12936 28815805

[92] Wang Z Yin W Zhu L Li J Yao Y Chen F. Iron drives T helper cell pathogenicity by promoting rna-binding protein Pcbp1-mediated proinflammatory cytokine production. Immunity (2018) 49(1):80–92.e7. doi: 10.1016/j.immuni.2018.05.008 29958803

[93] Vinuesa CG Linterman MA Yu D MacLennan IC. Follicular helper T cells. Annu Rev Immunol (2016) 34:335–68. doi: 10.1146/annurev-immunol-041015-055605 26907215

[94] Crotty S. T Follicular helper cell biology: A decade of discovery and diseases. Immunity (2019) 50(5):1132–48. doi: 10.1016/j.immuni.2019.04.011 PMC6532429 31117010

[95] Koutsakos M Wheatley AK Loh L Clemens EB Sant S Nussing S. Circulating T(Fh) cells, serological memory, and tissue compartmentalization shape human influenza-specific b cell immunity. Sci Transl Med (2018) 10(428):eaan8405. doi: 10.1126/scitranslmed.aan8405 29444980

[96] Deng J Chen Q Chen Z Liang K Gao X Wang X. The metabolic hormone leptin promotes the function of T(Fh) cells and supports vaccine responses. Nat Commun (2021) 12(1):3073. doi: 10.1038/s41467-021-23220-x 34031386 PMC8144586

[97] Yao Y Chen Z Zhang H Chen C Zeng M Yunis J. Selenium-Gpx4 axis protects follicular helper T cells from ferroptosis. Nat Immunol (2021) 22(9):1127–39. doi: 10.1038/s41590-021-00996-0 34413521

[98] Wang Y Tian Q Hao Y Yao W Lu J Chen C. The kinase complex Mtorc2 promotes the longevity of virus-specific memory Cd4(+) T cells by preventing ferroptosis. Nat Immunol (2022) 23(2):303–17. doi: 10.1038/s41590-021-01090-1 34949833

[99] Chen Z Wang N Yao Y Yu D. Context-dependent regulation of follicular helper T cell survival. Trends Immunol (2022) 43(4):309–21. doi: 10.1016/j.it.2022.02.002 35249831

[100] Hao Y Wang Y Liu X Yang X Wang P Tian Q. The kinase complex mtor complex 2 promotes the follicular migration and functional maturation of differentiated follicular helper Cd4(+) T cells during viral infection. Front Immunol (2018) 9:1127. doi: 10.3389/fimmu.2018.01127 29875775 PMC5974104

[101] Yang J Lin X Pan Y Wang J Chen P Huang H. Critical roles of mtor complex 1 and 2 for T follicular helper cell differentiation and germinal center responses. Elife (2016) 5:e17936. doi: 10.7554/eLife.17936 27690224 PMC5063587

[102] Zeng H Cohen S Guy C Shrestha S Neale G Brown SA. Mtorc1 and Mtorc2 kinase signaling and glucose metabolism drive follicular helper T cell differentiation. Immunity (2016) 45(3):540–54. doi: 10.1016/j.immuni.2016.08.017 PMC5050556 27637146

[103] Plitas G Rudensky AY. Regulatory T cells: Differentiation and function. Cancer Immunol Res (2016) 4(9):721–5. doi: 10.1158/2326-6066.CIR-16-0193 PMC5026325 27590281

[104] Newton R Priyadharshini B Turka LA. Immunometabolism of regulatory T cells. Nat Immunol (2016) 17(6):618–25. doi: 10.1038/ni.3466 PMC5006394 27196520

[105] Mougiakakos D Johansson CC Kiessling R. Naturally occurring regulatory T cells show reduced sensitivity toward oxidative stress-induced cell death. Blood (2009) 113(15):3542–5. doi: 10.1182/blood-2008-09-181040 19050306

[106] Xu C Sun S Johnson T Qi R Zhang S Zhang J. The glutathione peroxidase Gpx4 prevents lipid peroxidation and ferroptosis to sustain treg cell activation and suppression of antitumor immunity. Cell Rep (2021) 35(11):109235. doi: 10.1016/j.celrep.2021.109235 34133924

[107] Wang H Morse HC 3rd Bolland S. Transcriptional control of mature b cell fates. Trends Immunol (2020) 41(7):601–13. doi: 10.1016/j.it.2020.04.011 32446878

[108] Muri J Thut H Bornkamm GW Kopf M. B1 and marginal zone b cells but not follicular B2 cells require Gpx4 to prevent lipid peroxidation and ferroptosis. Cell Rep (2019) 29(9):2731–44.e4. doi: 10.1016/j.celrep.2019.10.070 31775041

[109] Clarke AJ Riffelmacher T Braas D Cornall RJ Simon AK. B1a b cells require autophagy for metabolic homeostasis and self-renewal. J Exp Med (2018) 215(2):399–413. doi: 10.1084/jem.20170771 29326381 PMC5789411

[110] Bailey AP Koster G Guillermier C Hirst EM MacRae JI Lechene CP. Antioxidant role for lipid droplets in a stem cell niche of drosophila. Cell (2015) 163(2):340–53. doi: 10.1016/j.cell.2015.09.020 PMC4601084 26451484

[111] Singh R Kaushik S Wang Y Xiang Y Novak I Komatsu M. Autophagy regulates lipid metabolism. Nature (2009) 458(7242):1131–5. doi: 10.1038/nature07976 PMC2676208 19339967

[112] Wang D Xie N Gao W Kang R Tang D. The ferroptosis inducer erastin promotes proliferation and differentiation in human peripheral blood mononuclear cells. Biochem Biophys Res Commun (2018) 503(3):1689–95. doi: 10.1016/j.bbrc.2018.07.100 PMC6179365 30049441

[113] Wang RN Green J Wang Z Deng Y Qiao M Peabody M. Bone morphogenetic protein (Bmp) signaling in development and human diseases. Genes Dis (2014) 1(1):87–105. doi: 10.1016/j.gendis.2014.07.005 25401122 PMC4232216

[114] Zhang Y Tan H Daniels JD Zandkarimi F Liu H Brown LM. Imidazole ketone erastin induces ferroptosis and slows tumor growth in a mouse lymphoma model. Cell Chem Biol (2019) 26(5):623–33.e9. doi: 10.1016/j.chembiol.2019.01.008 30799221 PMC6525071

[115] Schmitt A Xu W Bucher P Grimm M Konantz M Horn H. Dimethyl fumarate induces ferroptosis and impairs nf-Kappab/Stat3 signaling in dlbcl. Blood (2021) 138(10):871–84. doi: 10.1182/blood.2020009404 33876201

[116] Criscitiello MF Kraev I Lange S. Post-translational protein deimination signatures in serum and serum-extracellular vesicles of bos Taurus reveal immune, anti-pathogenic, anti-viral, metabolic and cancer-related pathways for deimination. Int J Mol Sci (2020) 21(8):2861. doi: 10.3390/ijms21082861 32325910 PMC7215346

[117] Mahajan A Herrmann M Munoz LE. Clearance deficiency and cell death pathways: A model for the pathogenesis of sle. Front Immunol (2016) 7:35. doi: 10.3389/fimmu.2016.00035 26904025 PMC4745266

[118] Li P Jiang M Li K Li H Zhou Y Xiao X. Glutathione peroxidase 4-regulated neutrophil ferroptosis induces systemic autoimmunity. Nat Immunol (2021) 22(9):1107–17. doi: 10.1038/s41590-021-00993-3 PMC8609402 34385713

[119] Gao X Song Y Wu J Lu S Min X Liu L. Iron-dependent epigenetic modulation promotes pathogenic T cell differentiation in lupus. J Clin Invest (2022) 132(9):e152345. doi: 10.1172/JCI152345 35499082 PMC9057600

[120] Anders HJ Saxena R Zhao MH Parodis I Salmon JE Mohan C. Lupus nephritis. Nat Rev Dis Primers (2020) 6(1):7. doi: 10.1038/s41572-019-0141-9 31974366

[121] Hong S Healy H Kassianos AJ. The emerging role of renal tubular epithelial cells in the immunological pathophysiology of lupus nephritis. Front Immunol (2020) 11:578952. doi: 10.3389/fimmu.2020.578952 33072122 PMC7538705

[122] Gomes MF Mardones C Xipell M Blasco M Sole M Espinosa G. The extent of tubulointerstitial inflammation is an independent predictor of renal survival in lupus nephritis. J Nephrol (2021) 34(6):1897–905. doi: 10.1007/s40620-021-01007-z 33721269

[123] Liu BC Tang TT Lv LL Lan HY. Renal tubule injury: A driving force toward chronic kidney disease. Kidney Int (2018) 93(3):568–79. doi: 10.1016/j.kint.2017.09.033 29361307

[124] Marks ES Bonnemaison ML Brusnahan SK Zhang W Fan W Garrison JC. Renal iron accumulation occurs in lupus nephritis and iron chelation delays the onset of albuminuria. Sci Rep (2017) 7(1):12821. doi: 10.1038/s41598-017-13029-4 28993663 PMC5634457

[125] Wlazlo E Mehrad B Morel L Scindia Y. Iron metabolism: An under investigated driver of renal pathology in lupus nephritis. Front Med (Lausanne) (2021) 8:643686. doi: 10.3389/fmed.2021.643686 33912577 PMC8071941

[126] Linkermann A Skouta R Himmerkus N Mulay SR Dewitz C De Zen F. Synchronized renal tubular cell death involves ferroptosis. Proc Natl Acad Sci U.S.A. (2014) 111(47):16836–41. doi: 10.1073/pnas.1415518111 PMC4250130 25385600

[127] Wang Y Bi R Quan F Cao Q Lin Y Yue C. Ferroptosis involves in renal tubular cell death in diabetic nephropathy. Eur J Pharmacol (2020) 888:173574. doi: 10.1016/j.ejphar.2020.173574 32976829

[128] Deane KD Holers VM. Rheumatoid arthritis pathogenesis, prediction, and prevention: An emerging paradigm shift. Arthritis Rheumatol (2021) 73(2):181–93. doi: 10.1002/art.41417 PMC7772259 32602263

[129] Smolen JS Aletaha D McInnes IB. Rheumatoid arthritis. Lancet (2016) 388(10055):2023–38. doi: 10.1016/S0140-6736(16)30173-8 27156434

[130] Nygaard G Firestein GS. Restoring synovial homeostasis in rheumatoid arthritis by targeting fibroblast-like synoviocytes. Nat Rev Rheumatol (2020) 16(6):316–33. doi: 10.1038/s41584-020-0413-5 PMC7987137 32393826

[131] Nemeth T Nagy G Pap T. Synovial fibroblasts as potential drug targets in rheumatoid arthritis, where do we stand and where shall we go? Ann Rheum Dis (2022) 81(8):1055–64. doi: 10.1136/annrheumdis-2021-222021 PMC9279838 35715191

[132] Ling H Li M Yang C Sun S Zhang W Zhao L. Glycine increased ferroptosis *Via* Sam-mediated Gpx4 promoter methylation in rheumatoid arthritis. Rheumatol (Oxford) (2022) 61(11):4521–34. doi: 10.1093/rheumatology/keac069 35136972

[133] Wu J Feng Z Chen L Li Y Bian H Geng J. Tnf antagonist sensitizes synovial fibroblasts to ferroptotic cell death in collagen-induced arthritis mouse models. Nat Commun (2022) 13(1):676. doi: 10.1038/s41467-021-27948-4 35115492 PMC8813949

[134] Ordas I Eckmann L Talamini M Baumgart DC Sandborn WJ. Ulcerative colitis. Lancet (2012) 380(9853):1606–19. doi: 10.1016/S0140-6736(12)60150-0 22914296

[135] Torres J Mehandru S Colombel JF Peyrin-Biroulet L. Crohn's disease. Lancet (2017) 389(10080):1741–55. doi: 10.1016/S0140-6736(16)31711-1 27914655

[136] Sartor RB Wu GD. Roles for intestinal bacteria, viruses, and fungi in pathogenesis of inflammatory bowel diseases and therapeutic approaches. Gastroenterology (2017) 152(2):327–39.e4. doi: 10.1053/j.gastro.2016.10.012 27769810 PMC5511756

[137] Gunther C Neumann H Neurath MF Becker C. Apoptosis, necrosis and necroptosis: Cell death regulation in the intestinal epithelium. Gut (2013) 62(7):1062–71. doi: 10.1136/gutjnl-2011-301364 22689519

[138] Werner T Wagner SJ Martinez I Walter J Chang JS Clavel T. Depletion of luminal iron alters the gut microbiota and prevents crohn's disease-like ileitis. Gut (2011) 60(3):325–33. doi: 10.1136/gut.2010.216929 21076126

[139] Kobayashi Y Ohfuji S Kondo K Fukushima W Sasaki S Kamata N. Association between dietary iron and zinc intake and development of ulcerative colitis: A case-control study in Japan. J Gastroenterol Hepatol (2019) 34(10):1703–10. doi: 10.1111/jgh.14642 30821862

[140] Chen Y Zhang P Chen W Chen G. Ferroptosis mediated dss-induced ulcerative colitis associated with Nrf2/Ho-1 signaling pathway. Immunol Lett (2020) 225:9–15. doi: 10.1016/j.imlet.2020.06.005 32540488

[141] Xu M Tao J Yang Y Tan S Liu H Jiang J. Ferroptosis involves in intestinal epithelial cell death in ulcerative colitis. Cell Death Dis (2020) 11(2):86. doi: 10.1038/s41419-020-2299-1 32015337 PMC6997394

[142] Wang S Liu W Wang J Bai X. Curculigoside inhibits ferroptosis in ulcerative colitis through the induction of Gpx4. Life Sci (2020) 259:118356. doi: 10.1016/j.lfs.2020.118356 32861798

[143] Mayr L Grabherr F Schwarzler J Reitmeier I Sommer F Gehmacher T. Dietary lipids fuel Gpx4-restricted enteritis resembling crohn's disease. Nat Commun (2020) 11(1):1775. doi: 10.1038/s41467-020-15646-6 32286299 PMC7156516

[144] Dodson M Castro-Portuguez R Zhang DD. Nrf2 plays a critical role in mitigating lipid peroxidation and ferroptosis. Redox Biol (2019) 23:101107. doi: 10.1016/j.redox.2019.101107 30692038 PMC6859567

[145] Adedoyin O Boddu R Traylor A Lever JM Bolisetty S George JF. Heme oxygenase-1 mitigates ferroptosis in renal proximal tubule cells. Am J Physiol Renal Physiol (2018) 314(5):F702–F14. doi: 10.1152/ajprenal.00044.2017 PMC6031916 28515173

[146] Lucchinetti CF Bruck W Rodriguez M Lassmann H. Distinct patterns of multiple sclerosis pathology indicates heterogeneity on pathogenesis. Brain Pathol (1996) 6(3):259–74. doi: 10.1111/j.1750-3639.1996.tb00854.x PMC7161824 8864283

[147] Rodriguez Murua S Farez MF Quintana FJ. The immune response in multiple sclerosis. Annu Rev Pathol (2022) 17:121–39. doi: 10.1146/annurev-pathol-052920-040318 34606377

[148] Voet S Prinz M van Loo G. Microglia in central nervous system inflammation and multiple sclerosis pathology. Trends Mol Med (2019) 25(2):112–23. doi: 10.1016/j.molmed.2018.11.005 30578090

[149] Hu CL Nydes M Shanley KL Morales Pantoja IE Howard TA Bizzozero OA. Reduced expression of the ferroptosis inhibitor glutathione peroxidase-4 in multiple sclerosis and experimental autoimmune encephalomyelitis. J Neurochem (2019) 148(3):426–39. doi: 10.1111/jnc.14604 PMC6347488 30289974

[150] Jhelum P Santos-Nogueira E Teo W Haumont A Lenoel I Stys PK. Ferroptosis mediates cuprizone-induced loss of oligodendrocytes and demyelination. J Neurosci (2020) 40(48):9327–41. doi: 10.1523/JNEUROSCI.1749-20.2020 PMC7687057 33106352

[151] Cui Y Zhang Z Zhou X Zhao Z Zhao R Xu X. Microglia and macrophage exhibit attenuated inflammatory response and ferroptosis resistance after Rsl3 stimulation *Via* increasing Nrf2 expression. J Neuroinflamm (2021) 18(1):249. doi: 10.1186/s12974-021-02231-x PMC8557003 34717678

[152] Lundberg IE Fujimoto M Vencovsky J Aggarwal R Holmqvist M Christopher-Stine L. Idiopathic inflammatory myopathies. Nat Rev Dis Primers (2021) 7(1):86. doi: 10.1038/s41572-021-00321-x 34857798

[153] Zanframundo G Tripoli A Cometi L Marcucci E Furini F Cavagna L. One year in review 2020: Idiopathic inflammatory myopathies. Clin Exp Rheumatol (2021) 39(1):1–12. doi: 10.55563/clinexprheumatol/qug8tf 32828143

[154] Lundberg IE de Visser M Werth VP. Classification of myositis. Nat Rev Rheumatol (2018) 14(5):269–78. doi: 10.1038/nrrheum.2018.41 29651121

[155] Shi J Tang M Zhou S Xu D Zhao J Wu C. Programmed cell death pathways in the pathogenesis of idiopathic inflammatory myopathies. Front Immunol (2021) 12:783616. doi: 10.3389/fimmu.2021.783616 34899749 PMC8651702

[156] Gono T Kawaguchi Y Hara M Masuda I Katsumata Y Shinozaki M. Increased ferritin predicts development and severity of acute interstitial lung disease as a complication of dermatomyositis. Rheumatol (Oxford) (2010) 49(7):1354–60. doi: 10.1093/rheumatology/keq073 20385617

[157] Rygiel KA Miller J Grady JP Rocha MC Taylor RW Turnbull DM. Mitochondrial and inflammatory changes in sporadic inclusion body myositis. Neuropathol Appl Neurobiol (2015) 41(3):288–303. doi: 10.1111/nan.12149 24750247 PMC4833191

[158] Boehler JF Horn A Novak JS Li N Ghimbovschi S Lundberg IE. Mitochondrial dysfunction and role of harakiri in the pathogenesis of myositis. J Pathol (2019) 249(2):215–26. doi: 10.1002/path.5309 PMC7219509 31135059

[159] Meyer A Laverny G Allenbach Y Grelet E Ueberschlag V Echaniz-Laguna A. Ifn-Beta-Induced reactive oxygen species and mitochondrial damage contribute to muscle impairment and inflammation maintenance in dermatomyositis. Acta Neuropathol (2017) 134(4):655–66. doi: 10.1007/s00401-017-1731-9 28623559

[160] Li C Deng X Xie X Liu Y Friedmann Angeli JP Lai L. Activation of glutathione peroxidase 4 as a novel anti-inflammatory strategy. Front Pharmacol (2018) 9:1120. doi: 10.3389/fphar.2018.01120 30337875 PMC6178849

[161] Li C Deng X Zhang W Xie X Conrad M Liu Y. Novel allosteric activators for ferroptosis regulator glutathione peroxidase 4. J Med Chem (2019) 62(1):266–75. doi: 10.1021/acs.jmedchem.8b00315 29688708

